# Dataset of geoelectrical properties for samples obtained at the Higashi-Aogashima Knoll Caldera hydrothermal field, Japan

**DOI:** 10.1016/j.dib.2025.111669

**Published:** 2025-05-15

**Authors:** Yusuke Ohta, Tatsuo Nozaki, Takafumi Kasaya

**Affiliations:** a Research Institute for Marine Resources Utilization, Japan Agency for Marine-Earth Science and Technology (JAMSTEC), 2-15 Natsushima-cho, Yokosuka, Kanagawa 237-0061, Japan; b Faculty of Science and Engineering, Waseda University, 3-4-1 Okubo, Shinjuku-ku, Tokyo 169-8555, Japan; c Frontier Research Center for Energy and Resources, School of Engineering, The University of Tokyo, 7-3-1 Hongo, Bunkyo-ku, Tokyo 113-8656, Japan

**Keywords:** Induced polarization, Resistivity, Porosity, Density, Seafloor massive sulfide deposit

## Abstract

This dataset contains fundamental measurements and analyses of rock samples collected from seafloor hydrothermal fields, with a focus on their physical and electrical properties. The dataset includes porosity and particle density measurements obtained via the buoyancy method and complex impedance data acquired through potentiostat-based electrochemical impedance spectroscopy with frequency sweeps. Additionally, impedance data provides Cole-Cole fitting analyses and corresponding graphical representations, which are essential for understanding the polarization characteristics of rock samples. Rocks in seafloor hydrothermal field often exhibit unique properties that are distinct from those of terrestrial rock physics models, necessitating specialized datasets for the accurate interpretation of seafloor geophysical and electromagnetic exploration. This dataset aims to bridge this gap by offering comprehensive data to aid in the interpretation of resistivity structures and improve rock physics models for seafloor hydrothermal systems. The data and figures provided herein are expected to facilitate advancements in the study of seafloor hydrothermal deposits and their resource potential.

Specifications Table

**Table:** 

Subject	Earth & Environmental Sciences
Specific subject area	Subseafloor resources engineering
Type of data	xlsx (porosity data, density data, Cole-Cole analyses results, descriptions) csv (Raw data and Pre-processed data for potentiostat electrochemical impedance spectroscopy data) Figure (frequency sweep graph, Cole-Cole fitting graphs, map)
Data collection	Rock samples were collected from the Higashi-Aogashima Knoll Caldera hydrothermal field during the cruise KM24-09 aboard the JAMSTEC research vessel Kaimei. Sampling was conducted within 32.436728°N–32.470738°N latitude and 139.866848°E–139.923142°E longitude using a manipulator of the remotely operated vehicle (ROV). Porosity and grain density were measured via the buoyancy method. Induced polarization data were obtained using potentiostat electrochemical impedance spectroscopy (EIS) over 0.1–10,000 Hz, under varied fluid conductivity conditions. Cole-Cole model analyses were performed to derive key parameters such as chargeability and relaxation time.
Data source location	Data source • Institution: Japan Agency for Marine-Earth Science and Technology (JAMSTEC) • City/Town/Region: Yokosuka, Natsushima-cho • Country: Japan • Location of samples • Latitude and longitude (and GPS coordinates, if possible) for collected samples/data: Lat. 32.436728°N-32.470738°N; Lon. 139.866848°E-139.923142°E*.*
Data accessibility	Repository name: Open science framework Data identification number: doi:10.17605/OSF.IO/W7JMA Direct URL to data: https://osf.io/w7jma/
Related research article	None.

## 1 Value of the Data

• The physical property data of rock samples from the target sea area and similar geological structures are beneficial for appropriately interpreting the geophysical exploration results. Such datasets offer ground truth that serves as a basis for interpretation in geophysical exploration studies of seafloor hydrothermal deposits.
• The measurement results of the complex impedance obtained through frequency sweep measurements, along with porosity and density data, can be utilized as a dataset for interpreting the results of electrical and electromagnetic surveys, particularly for shallow seabed layers. This dataset enables the precise tracking of changes in electrical properties across arbitrary frequency bands.
• The physical property parameters of the sample group contribute new data to cumulative studies of physical property data in the target sea area and seafloor hydrothermal fields of similar origin. Highly induced polarization samples from seafloor hydrothermal fields require expanded data for further study.

## 2 Background

This work provides a ground-truth dataset for geophysical exploration, targeting the Higashi-Aogashima Knoll Caldera hydrothermal field, Japan. Recent efforts to characterize the subsurface structure through marine electric and electromagnetic surveys have highlighted the increasing demand for reliable rock sample information. These surveys rely on the interpretation based on measurable rock properties. Although it is possible to estimate rock parameters by applying general rock properties derived from terrestrial rock physics studies, rock samples from seafloor hydrothermal fields often exhibit unique properties that do not conform to conventional rock physics models. Therefore, there is a continual demand for datasets that enable more direct and accurate interpretation by providing new data from actual rock samples. In this study, the porosity and particle density were measured using the buoyancy method and the complex impedance was measured via frequency sweeps. These datasets are expected to facilitate the accurate interpretation of geophysical exploration results and rock physics studies, not only in Higashi-Aogashima field but also across a wide range of seafloor hydrothermal systems. They will further contribute to metal resource exploration and research on the genesis of seafloor hydrothermal deposits and volcanogenic massive sulfide deposits on land.

## 3 Data Description

**Fig. 1:**
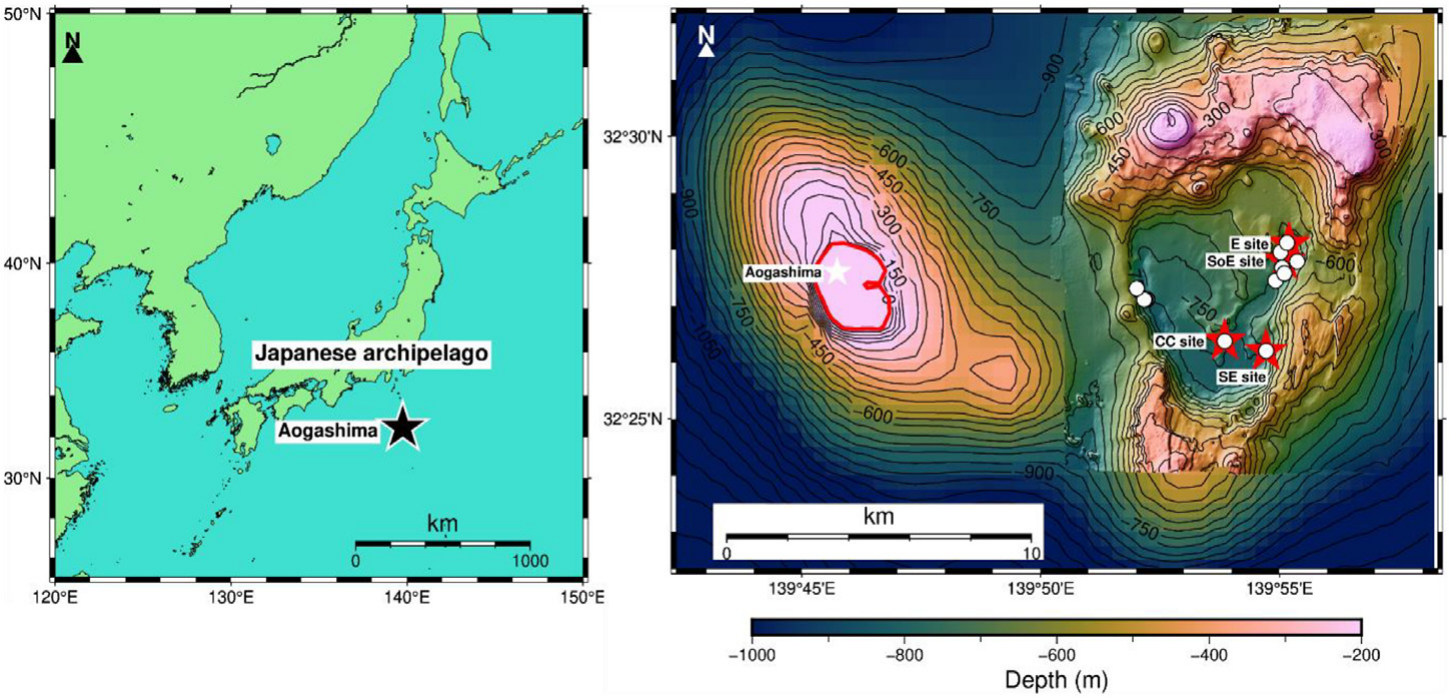
Composite figure showing (left) the location of Aogashima Island within the Japanese Archipelago and (right) the bathymetric map of the Higashi-Aogashima Knoll Caldera hydrothermal field. The bathymetry combines high-resolution multibeam echosounder (MBES) data with satellite-derived topography. Minor discontinuities in contour lines and shading are present at the junctions between datasets due to differences in spatial resolution and contour interpolation. The MBES data were obtained by R/V Yokosuka during cruise YK21-10 using a Kongsberg EM122 multi-beam echo sounder at a frequency of 12 kHz. Depths are shown relative to sea level (negative values), with contour intervals set at 50 meters. A continuous color gradient highlights depth variations across the caldera structure. Sampling locations are marked as white circles, and their precise geographic coordinates are listed in the accompanying dataset. Red star symbols indicate designated research sites: Central Cone (CC) site, Southeast (SE) site, East (E) site, and South of East (SoE) site.

This article describes the dataset of a linked repository [1] containing geophysical and rock physical data collected from the Higashi-Aogashima Knoll caldera hydrothermal field. The dataset was obtained during cruise KM24-09 [2] conducted by the Japan Agency for Marine-Earth Science and Technology (JAMSTEC) research vessel Kaimei. A total of 37 rock samples were collected, of which 17 were selected for repeated measurements to acquire the induced polarization data under multiple fluid conductivity conditions (Fig. 1).

The Higashi-Aogashima Knoll Caldera is located approximately 360 km south of downtown Tokyo and 12 km east of Aogashima Island. Detailed geological, topographical, and mineralogical information regarding this seafloor caldera is provided in [[3], [4], [5], [6]]. In addition, geophysical exploration efforts, have recently been initiated to better characterize the subsurface structure of this hydrothermal field (e.g., [7]) and require rock sample information. The caldera has a floor depth ranging from 600 m to 800 m and measures approximately 4.0 × 7.3 km in diameter. Sampling was conducted within the area bounded by min. Latitude: 32.436728°N, Max. Latitude: 32.470738°N, Min. Longitude: 139.866848°E, and Max. Longitude: 139.923142°E. All samples were hand-sampled from the seafloor using a manipulator of the remotely operated vehicle (ROV).

The porosities and particle densities of the collected samples were measured using the buoyancy method. In addition, the complex impedance was measured via a frequency sweep to obtain the induced polarization data. The measurements were conducted in multiple fluid-conductivity environments, which enabled a comprehensive understanding of the electrical properties of the samples. The measured impedance data were converted into complex conductivity data using a transformation based on the shape factor of the rock. To further enhance the usability of the data, additional data processing and analysis were performed using fundamental rock physics models. Users can easily obtain key parameter summaries from the fitting results of the multiple Cole–Cole model (CCM) [8], facilitating frequency-domain modeling of the electromagnetic properties of rocks in the seafloor hydrothermal field.

The dataset includes:

• This Excel file consists of three sheets: “Rock samples” contains fundamental physical properties such as sample ID, locality (latitude and longitude), lithology, porosity, and grain density; “CCM parameters” provides CCM fitting results, including low-frequency conductivity, chargeability, and time constant; “Comprehensive CCM” presents a comprehensive Cole-Cole model analysis based on [9]’s method, incorporating formation factor for parameter transformation. Each sheet organizes data from different perspectives on the physical and electromagnetic properties of the rock samples.
• Raw data holder: Contains all raw impedance data for each measurement, including repeated measurements.
• Standardized Data Folder: Normalized complex conductivity data for Cole–Cole analysis organized by sample. Data were standardized using the Savitzky-Golay method and averaged.
• Figure Folder: Storing graphical representations of standardized data, Cole-Cole fitting results. This folder also includes map figures.

This dataset offers a valuable resource for interpreting geophysical survey data from seafloor hydrothermal fields. This dataset supports research on rock physics, enhances the understanding of unique seafloor rock properties, and contributes to the exploration of metal resources and genesis of seafloor hydrothermal deposits.

## 4 Experimental Design, Materials and Methods

### 4.1 Rock samples

Rock samples were collected from the seafloor of the Higashi-Aogashima Knoll Caldera hydrothermal field within an area bounded by Min. Latitude: 32.436728°N, Max. Latitude: 32.470738°N, Min. Longitude: 139.866848°E, and Max. Longitude: 139.923142°E. All samples were hand-sampled at the seafloor using a manipulator of the ROV. Our 17 measured samples comprised six volcanic rocks, five sulfide-rich mound rocks, two volcanoclastic rocks, two dead sulfide-rich chimneys, an altered volcanic rock, and a sulfate-rich mound rock. The samples were cut into rectangular chips for the measurements, as detailed in the Induced Polarization Data section.

### 4.2 Porosity and grain density data

The saturation and buoyancy methods [10] were employed to determine the pore space and grain density of the samples. The buoyancy method calculates the pore space and grain density based on measurements of the wet weight in air ($W_{s}$), submerged weight in water ($W^{\prime }$), and dry weight ($W_{d}$). The weights are described by the following equations:

(1) $$W_{s}=V\left\{\phi \rho_{w}+\left(1-\phi \right)\rho_{d}\right\},$$

(2) $$W^{\prime }=V\left(1-\phi \right)\left(\rho_{d}-\rho_{w}\right),$$

(3) $$\;W_{d}=V\left(1-\phi \right)\rho_{d}$$

where ϕ is the porosity, V is the sample volume, $\rho_{d}$ is the grain density, and $\rho_{w}$ is the density of water. By solving these equations simultaneously, the porosity and grain density can be accurately determined.

### 4.3 Induced polarization data

Frequency sweep measurements such as Electrochemical Impedance Spectroscopy (EIS) were used to extract the induced polarization (IP) characteristics, including the critical frequency of polarization (threshold frequency of polarization). In the context of the Debye and Cole-Cole relaxation models, this corresponds to the reciprocal of the time constant. These measurements also reveal other material-related physical properties [[11], [12], [13]].

The effect of the IP was measured using EIS. The IP effect provides insight into material characteristics, particularly in rocks with high conductivity, owing to the presence of specific metallic minerals. A chemical impedance analyzer, IM3590 (HIOKI E.E. CORPORATION, Tokyo, Japan) was used for the measurements.

The samples were force-saturated with aqueous NaCl solution and analyzed for complex resistivity. The forced saturation procedure followed the guidelines established by [10]. Complex resistivity was measured using a quadrupole potentiostat for EIS over a frequency range of 0.1–10,000 Hz. The combination of fluid conductivities varied across the samples and is detailed in the respective sample files.

As the physical values obtained by EIS were complex impedances, the complex conductivity was extracted as a physical property using

(4) $$\sigma^{*}=\frac{L}{A}\cdot \frac{1}{Z^{*}},$$

where $\sigma^{*}$ and $Z^{*}$ indicate complex conductivity and complex impedance, respectively. L and A denote the length and cross-sectional area of the sample, respectively, as measured using the caliper method.

Repeated measurement data for the same rock under the same solution conductivity conditions were averaged as the standardized data. This averaging process ignores the phase reversal due to high-level noise and sets it to zero. The Savitzky-Golay filter [14] was also deployed for legal data to create standardized data, with 9 points of windows and 3-dimension assumption.

### 4.4 Cole-Cole analyses

The complex resistivity data obtained in section 2.3 were analyzed using the CCM [8]. The model parameters were determined by applying the nonlinear least-squares method to the complex conductivity data. The CCM equation is as follows:

(5) $$\sigma^{*}=\sigma_{0}\left[1+\left(\frac{m}{1-m}\right)\left\{1-\frac{1}{1+{\left(j\omega \tau \right)}^{c}}\right\}\right],$$

where m is the chargeability, represented by $\sigma_{\infty }$ (conductivity at very high frequency) and $\sigma_{0}$ (conductivity at very low frequency), as follows:

(6) $$m=\frac{\sigma_{\infty }-\sigma_{0}}{\sigma_{\infty }}.$$

In these equations, j is the imaginary unit and denotes the ω angular frequency; τ is a parameter called the central relaxation time, which is the reciprocal of the critical frequency of the IP effect; c (0≤c≤1) is the Cole–Cole exponent, which reflects the frequency range over which the IP effect occurs [15].

Because the complex conductivity data for this study had multiple phase peaks, we applied the following double-dispersion CCM [16,17], which is an extended CCM used when two dispersion components are present.

(7) $$\sigma^{*}=\sigma_{0}\left[1+\left(\frac{m_{1}}{1-m_{1}}\right)\left\{1-\frac{1}{1+{\left(j\omega \tau_{1}\right)}^{c_{1}}}\right\}\right]\left[1+\left(\frac{m_{2}}{1-m_{2}}\right)\left\{1-\frac{1}{1+{\left(j\omega \tau_{2}\right)}^{c_{2}}}\right\}\right],$$

Where the subscript in the parameters is an indicator that distinguishes different dispersion phenomena. The chargeability m of the entire sample was calculated as follows:

(8) $$m=1-\left(1-m_{1}\right)\left(1-m_{2}\right).$$

However, it should be noted that when calculating this chargeability, high-frequency charging components associated with Maxwell-Wagner polarization should be omitted from the analysis (for example, by not considering all charging units with τ less than τ < 10^−5^ as chargeability of the sample; see, e.g., [9]).

In addition to applying the CCM to each measurement dataset, the results of a comprehensive CCM analysis based on the method of [9] were also included. This was obtained through a simple unification approach, where $\sigma_{0}=F^{-1}\sigma_{w},$ with Fbeing the formation factor as defined by [18].

## Limitations

Raw data were obtained as laboratory-level measurements and contained low-level signal noise for all the samples. Noise caused by the settings and power supply of the measuring equipment was noticeable in some samples, and some of the data contained high-level noise that was difficult to analyze.

## Ethics Statement

The authors dully adhered to ELSEVIER ‘Ethics in publishing’ policy. There were no ethical issues associated with this study.

## CRediT Author Statement

**Yusuke Ohta***:* Methodology, Investigation, Formal analysis, Data curation, Validation, Writing –Review, & Editing; **Tatsuo Nozaki**: Investigation, Data curation; **Takafumi Kasaya**: Investigation, Data curation, writing –review, and editing.

## Data Availability

Open Science FrameworkDataset of geoelectrical properties for samples obtained at the Higashi-Aogashima Knoll Caldera hydrothermal field. (Original data)
